# Methylation Analyses in Liquid Biopsy of Lung Cancer Patients: A Novel and Intriguing Approach Against Resistance to Target Therapies and Immunotherapies

**DOI:** 10.3390/cancers17183021

**Published:** 2025-09-16

**Authors:** Domenico Trombetta, Marco Donatello Delcuratolo, Federico Pio Fabrizio, Francesco Delli Muti, Antonio Rossi, Antonella Centonza, Francesco Pio Guerra, Angelo Sparaneo, Michele Piazzolla, Paola Parente, Lucia Anna Muscarella

**Affiliations:** 1 Laboratory of Oncology, Fondazione IRCCS Casa Sollievo della Sofferenza, 71013 San Giovanni Rotondo, Italy; d.trombetta@operapadrepio.it (D.T.); f.dellimuti@operapadrepio.it (F.D.M.); fp.guerra@operapadrepio.it (F.P.G.); a.sparaneo@operapadrepio.it (A.S.); l.muscarella@operapadrepio.it (L.A.M.); 2 Unit of Oncology, Fondazione IRCCS Casa Sollievo della Sofferenza, 71013 San Giovanni Rotondo, Italy; donatello.m.delcuratolo@gmail.com (M.D.D.); antocento86@gmail.com (A.C.); 3 Department of Medicine and Surgery, University of Enna “Kore”, 94100 Enna, Italy; federicopio.fabrizio@unikore.it; 4 Oncology Centre of Excellence, Therapeutic Science & Strategy Unit, IQVIA, 20019 Milan, Italy; arossi_it@yahoo.it; 5 Unit of Thoracic Surgery, Fondazione IRCCS Casa Sollievo della Sofferenza, 71013 San Giovanni Rotondo, Italy; m.piazzolla@operapadrepio.it; 6 Unit of Pathology, Fondazione IRCCS Casa Sollievo della Sofferenza, 71013 San Giovanni Rotondo, Italy

**Keywords:** DNA methylation, biomarker, cell-free DNA, liquid biopsy, lung cancer, targeted therapy, immunotherapy

## Abstract

Background/Objectives: As one of the intensely studied epigenetic modifications, DNA methylation plays a key role in tumours, including lung cancer. Hypermethylation in tumour suppressor genes or hypomethylation in oncogenes is an important event in tumorigenesis. Minimally invasive detection of aberrant DNA methylation by liquid biopsy could provide invaluable biomarkers for lung cancer screening, early diagnosis, prognosis, and treatment, also providing a useful tool to monitor response to targeted therapies and immunotherapies. Methods: Here, we discuss the current state-of-the-art cfDNA methylation analysis of NSCLC patients, examine the unique features and limitations of detection methods in a clinical context, and highlight the last research findings in the context of main biological and immunological therapies in lung cancer. Thus, the main goal of this review is to provide a critical overview of the most important published studies that report cfDNA methylation as prognostic biomarker for resistance to target therapies and immunotherapies in lung cancer. Results and Conclusions: DNA methylation-based biomarkers show promise for lung cancer detection and management. In particular, ctDNA methylation has been shown to play an important role in detecting resistance to tyrosine kinase inhibitors and immunotherapies. Nonetheless, DNA methylation biomarkers still lack large-scale validation, actually precluding their rapid implementation in clinical practice.

## Simple Summary

DNA methylation is a key event in the onset and progression of tumours, including lung cancer. Recently, the ability to identify and measure this epigenetic modification in patients’ circulating DNA has opened new avenues for early detection of cancer, prognosis, and the ability to minimally invasively monitor patient response to targeted therapies and immunotherapies. This review summarizes the main methodological approaches for studying DNA methylation in liquid biopsy and its potential use as novel biomarkers in precision medicine applied to lung cancer patients. While promising, this promising non-invasive approach still needs large-scale validation before DNA methylation can be widely used in clinics.

## 1. Introduction

Epigenetic mechanisms include DNA methylation, histone modifications, and non-coding RNAs regulation. Aberrant DNA methylation is the most common molecular lesion of the cancer cell. Methylation is an incorporation of a methyl group (-CH3) into a cytosine in regions enriched with CG bases, also known as CpG islands [1]. Methylation occurs in CpG islands when found at the promoter regions of several genes, and it often results in gene silencing, commonly found in tumour suppressor genes and linked to many neoplastic processes [2]. On the other hand, the transcriptional activation of genes with the methylation present in the gene body is associated with various cancer types, including lung cancer [3]. Methylome analysis has yielded highly successful results on tumour tissues, especially when the analysis is focused on molecular subtyping and biomarkers discovery of several tumour types, including lung cancer [4]. Though invasive methods such as tissue biopsy are considered the gold standard for diagnosis and disease monitoring, they have several limitations. DNA methylation has been recently detected in circulating cell-free DNA (ccfDNA) and circulating tumour DNA (ctDNA) and actually, it is considered an intriguing biomarker for translation into clinical applications to be used for diagnosis, prognosis, and predictive purposes [5,6].

One of the latest challenging issues actually remaining is how methylation in cfDNA correlates with targeted therapies and immunotherapy resistance in lung cancer patients [7].

## 2. Aberrant DNA Methylation in Cancer

DNA methylation involves the addition of a methyl group (-CH3) to a DNA molecule, usually at the level of cytosines, when they are followed by a guanine (CpG dinucleotide). A family of DNA methyltransferase enzymes (DNMTs) catalyses the process. While DNMT3A/DNMT3B are involved in de novo methylation, DNMT1 is in charge of preserving methylation patterns during DNA replication. However, DNMT1 has also been shown to undertake de novo DNA methylation, while DNMT3a and DNMT3b methyltransferases have been described to perform maintenance methylation as well [8]. This process is typically well-studied in promoter areas known as CpG islands, which contain a significant number of clustered CpG dinucleotides, the main target for DNA methylation.

In addition to preventing transcription factors (TFs) from binding to DNA sequences and hence influencing gene transcription, DNA methylation can alter chromatin structure and induce methylated binding protein (MBP) to bind to transcription inhibitors [9]. Prior research has shown that gene transcription is suppressed by hypermethylation in promoters. On the other hand, promoter hypomethylation encourages gene transcription. Regarding the gene’s body, transcription may be aided by DNA methylation in the gene body [10]. The distribution of methylation in the genome is bimodal; in general, the majority of regions are highly methylated (>85%), while <5% of CpG islands are unmethylated [11,12]. Numerous genes, including those that are uniquely expressed in particular tissues, are found in the methylated fraction. Conversely, genes with CpG island promoters remain constitutively unmodified, mostly for housekeeping functions [13]. Even in tissues where the corresponding genes are not expressed, nearly all CpG islands are unmethylated. Despite this, in cancer and worldwide DNA hypomethylation, DNA hypermethylation occurs at several CpG islands [14]. Tumorigenesis is linked to either hypomethylation in oncogenes or hypermethylation in tumour suppressor genes [15].

The characteristic of cancer cells is the development of an abnormal DNA methylation pattern. Despite evidence of regional hypermethylation, DNA methylation has been proposed as a contributing factor to the development of cancer. In fact, it has been discovered that cancers have 5–10% lower global levels of 5-methylcytosine (5mC) than normal cells [16,17,18]. Tumour suppressor genes (TSGs) and other transcriptional regulatory elements, including gene promoters and enhancers, are commonly shown to be hypermethylated in cancer cells [19]. Specifically, the identification of hypermethylation in TSG promoter areas raises the possibility that epimutation serves as an oncogenesis-promoting agent.

Many cancer suppressor genes whose gene expression is silenced by DNA hypermethylation have been found in tumour tissues [20], with the first example being the discovery of DNA methylation in the promoter region of the retinoblastoma tumour suppressor gene (RB1) in patients with retinoblastoma [21]. Both germline mutation in familial malignancies and DNA hypermethylation in these genes are tissue-specific [22]. Hypomethylation of DNA throughout the genome is another trait of cancer cells. It causes abnormal gene expression [23,24], the activation of imprinted genes and retrotransposons, and chromosomal instability [25,26]. DNA hypermethylation is usually observed at megabase-scale DNA blocks known as partly methylated domains (PMDs), according to numerous studies employing genome-wide sequencing [27,28,29]. About half of the genome is made up of PMDs, which are characterized by a repressive chromatin structure linked to nuclear lamina, delayed replication, and high rates of somatic mutations [30,31].

## 3. cfDNA Methylation Analysis Methods

DNA methylation profiling has become a cornerstone of cancer epigenetics, offering valuable insights into tumour biology and enabling the development of minimally invasive biomarkers through the analysis of cfDNA [32].

Actually, this profiling primarily relies on two methodological approaches: bisulfite-based and bisulfite-free, each offering distinct advantages depending on sample type and clinical application. Bisulfite conversion-based methods have long been the gold standard for DNA methylation analysis due to their ability to provide single-base resolution, enabling precise detection of methylation status at individual cytosine residues [33]. Alongside these methods, bisulfite-free strategies are emerging as promising applications, leveraging enrichment or restriction enzyme-based strategies, to preserve DNA integrity while maintaining sufficient sensitivity for cfDNA analysis [34].

These methodological advances have expanded the clinical potential of cfDNA methylation profiling for early cancer detection, treatment monitoring, and tumour origin and classification across multiple solid cancer types [35].

### 3.1. Bisulfite Conversion-Cased Methods

Bisulfite conversion is a critical step in DNA methylation assessment, providing nucleotide-level resolution by converting unmethylated cytosines to uracils while preserving methylated cytosines [36]. When integrated with high-throughput sequencing, this approach allows comprehensive analysis of methylation patterns across multiple genomic sites, thus facilitating the identification of novel epigenetic biomarkers [37].

For tissue-based studies, commonly used high-resolution bisulfite conversion-based methods include whole-genome bisulfite sequencing (WGBS) and reduced representation bisulfite sequencing (RRBS), which are commonly applied in solid tumours such as lung, breast, liver, and colorectal tumours and melanoma in order to investigate CpG-rich methylation regions [33]. WGBS provides the most exhaustive coverage, capturing methylation not only in CpG sites but also in non-CpG dinucleotides such as CpA, CpT, and CpC [38]. While traditionally requiring large amounts of intact DNA, recent methodological advances have lowered input amounts, enabling WGBS to be applied to low-input samples, including cfDNA from breast cancer, hepatocellular carcinoma (HCC), and lung cancer at early and advanced stages [39,40]. However, bisulfite treatment is inherently harsh, often leading to substantial DNA degradation, which compromises its sensitivity in the context of liquid biopsy and limits its utility for fragmented cfDNA [41].

To mitigate these challenges, RRBS is considered a cost-efficient alternative that uses MspI digestion to enrich CpG-dense regions, achieving focused methylome coverage and significantly reducing sequencing costs [42]. Nevertheless, its reliance on high-quality input DNA similarly restricts its applicability to cfDNA (cfRRBS), as well as reported in invasive epithelial ovarian cancer (EOC) and invasive ductal breast cancer (IDBC) [43,44].

To overcome such limitations, alternative approaches like methylated CpG tandem amplification and sequencing (MCTA-Seq) have been applied to plasma samples from gastric cancer (GC) patients, successfully identifying promising cfDNA methylation biomarkers for non-invasive GC detection [45]. Similar results were reported in HCC, where hypermethylated CpG islands in cfDNA were higher in HCC patients compared with healthy individuals [46]. For applications that are based on ultra-low input and high sensitivity, MCTA-Seq provides an effective solution by performing methylation detection from low quantity of DNA through the targeting of hypermethylated CGGCGG motifs, although this sequence-specific focus inherently restricts the range of regions that can be analysed [46].

Among PCR-based assays, quantitative methylation-specific PCR (qMSP) is one of the first methods to demonstrate that an increased promoter methylation levels within TSGs region could be detected in cfDNA from lung cancer patients, and it has since been widely applied across multiple tumour types with the aim of detecting methylation-based biomarkers [33,47].

Pyrosequencing and droplet digital methylation-specific PCR (ddMSP) are essential and common methods for analysing DNA methylation status in cfDNA. Pyrosequencing operates on single-stranded DNA, while ddMSP targets double-stranded DNA, both providing high sensitivity, making them well-suited for low-input samples, and offering cost-effective solutions. Although pyrosequencing is widely used, its limited sensitivity (>5% methylated alleles) restricts its ability to detect low-abundance tumour DNA; nonetheless, it has successfully identified promoter methylation in plasma from patients with diffuse large B-cell lymphoma (DLBCL) and glioblastoma, including O6-methylguanine-DNA methyltransferase (MGMT) methylation in cases where tumour tissue was unavailable [48,49].

In the same way, ddMSP, with higher sensitivity, enables detection of tumour-specific mutations like tumour protein p53 (TP53) in low-level ctDNA from HNSCC patients, supporting its role in post-treatment monitoring and early relapse detection [50].

Using plasma cfDNA samples from advanced colorectal cancer, NSCLC, breast cancer, or melanoma patients, targeted bisulfite methylation sequencing of 9223 CpG sites accurately detected the presence and type of cancer and this method allows for molecular testing of liquid biopsies regardless of specific oncogenic mutations [51].

Interestingly, Methyl-Seq is an innovative, cost-effective method for profiling the cell-free DNA methylome (cfMethyl-Seq), providing robust CpG island enrichment and precise detection of the tissue of origin for various cancers, including colorectal, liver, lung, and gastric cancers [52]. It has also been utilized to develop one of the first models for non-invasive lung cancer subtyping, offering a cost-effective strategy to capture genome-wide epigenetic signatures while overcoming challenges associated with bisulfite-induced DNA loss and fragmentation, thus enabling more accurate and reliable detection of methylation patterns in cfDNA for cancer classification and early detection [53]. Through the integration of enzymatic dephosphorylation and dideoxynucleotides during the end-repair process, cfMethyl-Seq preserves the integrity of cfDNA during library preparation. As a result, the overall sensitivity and accuracy of methylation detection can be compromised, particularly with low-input or fragmented cfDNA samples [52,54].

Notably, high-throughput methylation arrays such as the Epigenome-wide Profiler Illumina Human Methylation 450K (HM450K) and the Illumina Infinium Human MethylationEPIC (HM850K) BeadChip significantly broaden the analysis by profiling approximately 450,000 and 850,000 CpG sites, respectively, and are extensively used to assess comprehensive methylation landscapes across diverse genomic regions in multiple solid tumours grouping lung cancer, providing broad coverage of CpG sites but still lacking genome-wide depth and requiring substantial DNA input [55]. Despite this, these platforms require a substantial amount of input DNA, which limits their effectiveness for cfDNA-based profiling, as circulating free DNA is typically present in low quantities in plasma or serum samples, making the analysis more challenging [37]. Using serum cfDNA, these arrays successfully captured genome-wide methylation patterns that distinguish healthy individuals from those with advanced colorectal neoplasia, highlighting the potential of cfDNA as a comprehensive epigenomic resource for non-invasive biomarker discovery and early colorectal cancer detection [56].

An enrichment-based method targeting CpG-rich regions, Heatrich-bisulfite sequencing (Heatrich-BS), combined with a dedicated bioinformatics algorithm, enables precise tumour burden estimation, treatment response monitoring in colorectal cancer patients, and epigenetic subtyping to support patient stratification, thus offering a non-invasive and cost-effective solution [57].

Targeted bisulfite sequencing methods, such as bisulfite amplicon sequencing and bisulfite padlock probe sequencing (BSPP), enable high-resolution analysis of specific loci in various tumours. Particularly, BSPP has shown promise in cfDNA applications for the early detection of liver and colorectal cancers [58], as well as in the analysis of HCC tumour DNA cohorts and their matched plasma ctDNA [59].

All these approaches are needed for the careful and precise optimization of the assay parameters, including primer design, reaction conditions, and amplification protocols, to minimize the risk of amplification bias and ensure reliable and reproducible performance across a wide range of genomic targets.

### 3.2. Bisulfite Conversion-Free Methods

Bisulfite-free enrichment-based methods following antibody-based enrichment or restriction enzyme-based strategies, are more recently used and allow methylation profiling without the need for bisulfite treatment [37].

Among these, methylated DNA immunoprecipitation sequencing (MeDIP-seq) and its liquid biopsy adaptation, cell-free methylated DNA immunoprecipitation sequencing (cfMeDIP-seq), are enrichment-based methods for genome-wide DNA methylation profiling that capture methylated DNA fragments, enabling non-invasive detection of cancer-specific differentially methylated regions (DMRs). This approach has proven effective in cfDNA analysis, with several studies identifying hypermethylated promoter regions in plasma cfDNA with minimal input as potential early diagnostic markers for lung cancer, renal cell carcinoma, and pancreatic adenocarcinoma patients [60,61,62].

An alternative enrichment-based strategy, known as methyl-CpG-binding domain sequencing (MBD-seq), utilizes MBD proteins to selectively isolate methylated DNA fragments, with a particular focus on CpG-dense regions. Its cfDNA-adapted form, cfMBD-seq, requires minimal DNA input and has demonstrated diagnostic performance comparable to WGBS, with successful applications in cfDNA from plasma or serum in multiple cancer types, including colorectal, pancreatic, lung and breast cancers [63]. The DMRs detected through this strategy showed robust performance in the discrimination of cancerous samples, highlighting its effectiveness in identifying early-stage and metastatic prostate cancers [64]. Moreover, MBD-enrichment next generation sequencing (NGS) methods have been explored specifically for small cell lung cancer (SCLC), showing better sensitivity than low-pass whole-genome sequencing for detecting methylation signals in cfDNA [65].

In addition, 5-hydroxymethylcytosine (5hmC) in cfDNA has emerged as a promising biomarker for the non-invasive early detection of acute myeloid leukaemia (AML) patients [66]. Unlike 5mC, which acts as a repressive epigenetic mark, 5hmC provides distinct regulatory information with lower background noise. The 5hmC-Seal technique, which employs β-glucosyltransferase to label 5hmC-containing DNA with biotin for subsequent enrichment and sequencing, allows sensitive and genome-wide profiling with minimal DNA damage [35]. It requires low DNA input and has been successfully applied in early-stage cancers such as glioblastoma and lung cancer [67,68]. Furthermore, cancer-specific 5hmC signatures in cfDNA have shown high discriminatory power across multiple tumour types, including colorectal, gastric, pancreatic, liver, and thyroid cancers [69]. Despite its sensitivity and specificity, the multi-step protocol limits its adaptability for high-throughput or routine clinical applications.

Several NGS-based methods are carried out to analyse cfDNA methylation, with methylation-sensitive restriction enzyme (MRE)-seq demonstrating potential for detecting global hypomethylation patterns in liquid biopsy samples in diagnosis of solid cancer such as colorectal and lung, as well as predicting their cancer signal origin. The ability of this method to detect early-stage cancers is likely attributed to its preservation of DNA integrity, achieved by avoiding bisulfite conversion, and its selective enrichment of demethylated cancer-specific regions [70]. Among these, the HELP (HpaII tiny fragment enrichment by ligation-mediated PCR) assay provides locus-specific, genome-wide methylation profiling, analysing small DNA fragments generated through HpaII digestion, and has shown that lower global DNA methylation levels of long interspersed nuclear element-1 (LINE-1) prior to treatment are associated with poorer overall survival (OS) in GC patients [71].

Enrichment-based methods are complemented by highly sensitive assays such as digital droplet PCR (ddPCR) and quantitative PCR (qPCR), which are ideal for available DNA low-input, locus-specific analysis of pre-identified methylation markers in cfDNA as cost-effective tools for multiple cancer detection, including colorectal, lung, breast, and prostate cancers [33,72,73]. However, their reliance on known methylation markers limits their ability to detect novel methylation patterns and may yield false positives or negatives depending on the methylation density of the target regions [74].

On the other side, qPCR assays are designed to target specific methylation region at short stature homeobox gene 2 (SHOX2) and prostaglandin E receptor 4 (PTGER4) genes in plasma cfDNA with the aim of demonstrating strong potential for early lung cancer detection [75]. Moreover, increased cfDNA levels and hypermethylation of CpG islands occurred as Ras-association domain family member 1A (RASSF1A), fragile histidine triad (FHIT), adenomatous polyposis coli (APC), and glutathione S-transferase Pi 1 (GSTP1) genes in plasma, and they have emerged as promising non-invasive diagnostic biomarkers, confirming their utility in detecting renal and prostate cancers and correlating methylation patterns with disease progression [64,76]

**Figure 1:**
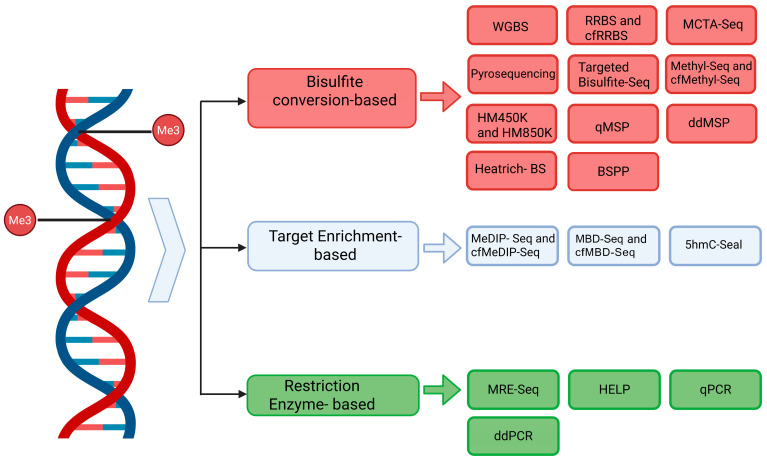
cfDNA methylation analysis methods. The workflow for cfDNA methylation analysis from blood samples typically begins with peripheral blood collection, followed by plasma separation through centrifugation. Circulating cfDNA is then extracted from the plasma and subjected to methylation profiling using one of several approaches, including bisulfite conversion-based methods, target enrichment techniques, or restriction enzyme-based strategies. Created in BioRender. Guerra, F. (2025) https://BioRender.com/h0830hg, accessed on 25 June 2025. Abbreviations. On the top: Me3, methyl groups (–CH_3_); WGBS, whole-genome bisulfite sequencing; RRBS, reduced representation bisulfite sequencing; cfRRBS, cell-free reduced representation bisulfite sequencing; MCTA-seq, methylated CpG tandem amplification and sequencing; targeted bisulfite-Seq, targeted bisulfite sequencing; Methyl-Seq, methylation sequencing; cfMethyl-seq, cell-free methylation sequencing; HM450K, HumanMethylation450 BeadChip; HM850K, MethylationEPIC BeadChip; qMSP, quantitative methylation-specific PCR; ddMSP, digital droplet methylation-specific PCR; Heatrich-BS, Heatrich-bisulfite sequencing; BSPP, bisulfite padlock probe sequencing. In the middle: MeDIP-seq, methylated DNA immunoprecipitation sequencing; cfMeDIP-seq, cell-free methylated DNA immunoprecipitation sequencing; MBD-seq, methyl-CpG-binding domain sequencing; cfMBD-seq, cell-free methyl-CpG-binding domain sequencing; 5hmC-Seal, 5-hydroxymethylcytosine Seal. At the bottom: MRE-Seq, methylation-sensitive restriction enzyme-Sequencing; HELP, HpaII tiny fragment enrichment by ligation-mediated PCR; qPCR, quantitative polymerase chain reaction; ddPCR, digital droplet polymerase chain reaction.

Figure 1 illustrates the primary methodologies used in cfDNA methylation analysis, categorized based on different conversion systems as well as bisulfite conversion-based methods and bisulfite-free approaches (Figure 1).

Table 1 summarizes the most widely used techniques, highlighting their underlying technologies, targets, advantages, limitations, and DNA input requirements.

Although bisulfite-based methods continue to be invaluable for generating high-resolution, single-base methylation maps, bisulfite-free approaches offer distinct benefits, particularly in preserving DNA integrity and enabling the analysis of low-input, fragmented cfDNA. These innovations hold significant potential to revolutionize early cancer detection, improve patient prognosis, and expand the clinical utility of ctDNA-based diagnostic approaches [33].

Overall, the integration of both bisulfite-dependent and bisulfite-free methylation profiling strategies provides a robust and complementary framework to enhance the precision, sensitivity, and clinical applicability of epigenetic biomarkers across a broad range of cancer types and biological samples.

## 4. cf/ctDNA Origin and Clinical Applications in Lung Cancer

Cell-free DNA (cfDNA) is a mixture of fragmented of DNA molecules originating from various tissues throughout the body. Its presence in the bloodstream was first reported by Mandel and Metais in 1948 [78].

**Figure 2:**
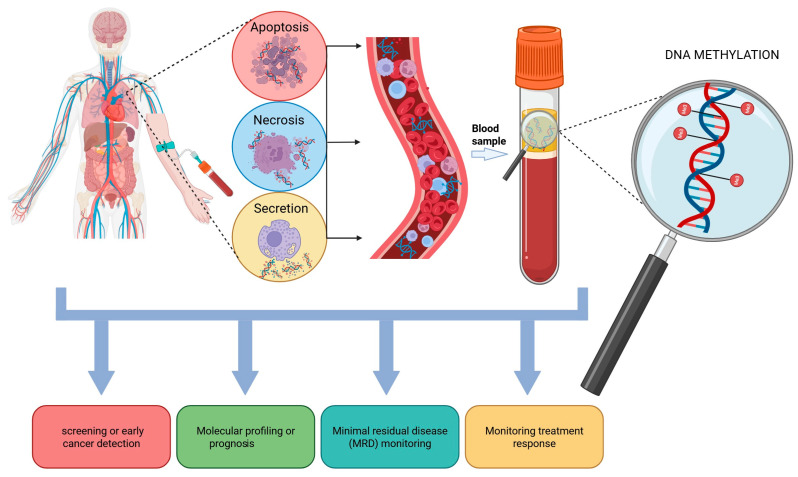
cf/ctDNA origin and its clinical applications. ctDNA is released in the bloodstream by apoptotic or necrotic tumour cells; it can be isolated from plasma and then analysed to identify DNA methylation changes. ctDNA methylation markers can provide information on early detection/screening, molecular profiling and prognosis, the monitoring of minimal residual disease or therapy response. Created in BioRender. Guerra, F. (2025) https://BioRender.com/5369f13, accessed on 25 June 2025.

In healthy individuals, cfDNA is released into the bloodstream, with hematopoietic cell turnover being proposed as the primary source, largely linked to cell death through apoptosis [79,80]. During apoptosis, cells undergo a systematic process of disassembly, during which nuclear DNA is fragmented into segments, generally about 150–200 base pairs long, matching the length of DNA wound around a nucleosome [81]. Cellular components, like nucleic acids, are subsequently encapsulated into apoptotic bodies, shielding them from degradation by circulating nucleases. These apoptotic bodies are removed through phagocytosis, enzymatically broken down, and released as soluble debris; however, a fraction may evade this process and enter the bloodstream as circulating cell-free DNA (cfDNA) [82,83]. Compared with healthy individuals, cancer patients’ blood displays significantly elevated levels of circulating cell-free DNA (cfDNA) [84]. This phenomenon was first reported in 1977 by Leon and colleagues [85], although it was later linked to the death of neoplastic cells [86]. Apoptosis and necrosis, two processes stemming from hypoxic and metabolic stress associated with cancer, are key contributors to the release of circulating tumour DNA (ctDNA) into the bloodstream (Figure 2). Unlike apoptosis, which plays a role in eliminating abnormal or redundant cells in a controlled manner, necrosis—identified as a major source of cfDNA in cancer patients—serves as a rapid and direct reflection of the adverse tumour microenvironment. Necrotic cells experience organelle dysfunction and plasma membrane damage, which can lead to the unregulated release of cellular components, exposing tumour DNA to degradative agents such as nucleases and free radicals [87]. Due to this uncoordinated release and digestion of DNA during necrosis, larger DNA fragments, often several kilobase pairs (kbp) in size, are thought to be released into circulation [82,88]. The distinct size profile of these fragments serves as an important characteristic for identifying necrosis-derived cfDNA [82]. The process of ctDNA release due to necrosis is complex, as necrotic cancer cells not only produce immune cell attractants but are also swiftly cleared—along with their leaked contents—primarily by macrophages. This clearance involves the digestion of cellular DNA, leading to the subsequent release of partially degraded ctDNA into the extracellular space [89,90].

Genetic and epigenetic features of ctDNA molecules can reflect the genome or epigenome structure of the cell of origin. The concentration of cfDNA in healthy adults is generally very low, often less than 10 ng per ml of plasma [91]. Elevated levels of cfDNA can be detected under many conditions, apart from cancer, comprising trauma, inflammation, myocardial infarction, stroke, sepsis, and chronic diseases such as diabetes mellitus but also physiological conditions as pregnancy. Particularly, in pregnant women, foetal cfDNA is released into the maternal circulation from placental trophoblasts. Similarly, cfDNA from transplanted organs (donor-derived cfDNA) appears in recipients’ blood post-transplant. The cfDNA concentration in cancer patients can be anywhere from the normal range to 50 times the normal levels, and ctDNA ever represents a small fraction (often <1%) of the total cfDNA in the blood. ctDNA can be identified in nearly all cases of some types of certain cancer types, such as bladder, colorectal, and ovarian cancer. For most other cancer types, it can be identified in over half of the cases, while detection rate for gliomas can be as low as 10% [92,93].

The concentration of circulating tumour DNA (ctDNA) in plasma, as well as the proportion of patients with detectable ctDNA levels, has been found to align closely with tumour stage. Typically, higher ctDNA concentrations are observed in advanced or metastatic diseases. For instance, a study involving 640 patients across various cancer types and stages reported a dramatic increase–of up to 100-fold–in median ctDNA concentrations for stage IV patients compared with those in stage I. The detection rates for ctDNA were 47%, 55%, 69%, and 82%, corresponding to stages I through IV of cancer progression, respectively [94]. In terms of its half-life in the bloodstream, ctDNA remains relatively short-lived, ranging from 16 min to approximately 2.5 h [95,96]. Multiple factors govern the concentration and persistence of ctDNA, offering valuable insights into real-time tumour burden. These include tumour characteristics such as volume, type, location, vascularization, and whether ctDNA is encapsulated within vesicles or bound to protein complexes. Additionally, therapeutic interventions—such as surgery, chemotherapy, or radiotherapy–and hepatic or renal clearance can further impact ctDNA dynamics [82,95]. Clearance and degradation processes are also influenced by interactions between circulating free DNA (cfDNA) molecules and serum proteins or protein complexes. Studies indicate that these macromolecular formations, which may consist of cfDNA alongside entities like monoclonal antibodies, albumin, or nucleosomes, hinder access to DNases and consequently slow cfDNA degradation [97]. Moreover, cfDNA uptake by cells—whether through adsorption to cell surface proteins or internalization across cell membranes—has been proposed as another mechanism affecting cfDNA clearance [96,98].

Because of in cancer patients ctDNA originate from malignant cells and carries tumour-specific genetic and epigenetic alterations, the tumour-derived fragments typically reflect the heterogeneity of the tumour and can provide a real-time snapshot of its molecular profile. The use of cfDNA as a surrogate for tumour tissue in cancer diagnostics offers several advantages. It enables non-invasive sampling through liquid biopsy, which can be repeated over time to monitor treatment response; detects minimal residual disease; and identifies emerging resistance mutations. Furthermore, cfDNA analysis can capture spatial and temporal tumour heterogeneity that may be missed by single-site tissue biopsies. However, there are important limitations. The concentration of ctDNA within total cfDNA can be exceedingly low, especially in early-stage cancers or in tumours with low turnover, posing technical challenges for detection and increasing the risk of false negatives. Additionally, biological and pre-analytical variables—such as individual physiology, disease state, DNA degradation, clearance rate, and sample handling—can affect the reliability of results. Despite these challenges, ctDNA remains a powerful and increasingly validated biomarker, with growing utility in personalized oncology and disease monitoring [33,99].

### ctDNA Methylation as Biomarkers in Lung Cancer

ctDNA represents a promising biomarker that carries cancer-specific genetic and epigenetic aberrations. As a result, it can be utilized as a substitute of tumour DNA in cancer diagnosis and prognosis prediction [100]. Actually, ctDNA analysis is mainly focused on the detection of cancer-specific mutations that are very important for therapeutic treatment and monitoring of cancer patients [80]. However, ctDNA methylation analysis seems to be one of the most potential ways among all liquid biopsy applications in cancer patients and can open new scenarios in the field of non-invasive oncological diagnostics (Figure 2) [101].

The scientific literature is full of evidences showing that aberrant DNA methylation contributes to tumorigenesis and tumour progression, principally through global hypomethylation, focal hypermethylation at multiple genomic regions (mostly CpG islands), and direct mutagenesis at methylated cytosines [102]. Compared with other classes of molecular biomarkers, the DNA methylation of CpG islands is highly in tumours and its measurements at multiple gene locus could also contribute to among various histological as well as molecular cancer subtypes [103].

The advantages of using ctDNA methylation as a liquid biomarker can be summarized in two key areas: sensitivity and specificity. Methylation analysis has demonstrated exceptional sensitivity and specificity in detecting tumour recurrence and monitoring disease progression. Notably, some studies have found that minimal residual disease (MRD) detection based on methylation is significantly more sensitive than methods relying on ctDNA mutations [101]. This highlights the potential of methylation analysis to identify patients with tumours more accurately, facilitating earlier diagnosis and intervention [101]. Additionally, the methylation patterns of cfDNA are highly consistent with the cells or tissues they originate from [104]. As a result, detecting tumour-specific DNA methylation abnormalities in patient plasma could serve as a promising avenue for developing blood-based tests aimed at early cancer diagnosis and prognosis. Furthermore, this approach may help pinpoint the tissue of origin for tumours, enhancing diagnostic precision and treatment planning [105,106,107,108,109].

Changes in DNA methylation patterns in plasma are known to arise early during cancer pathogenesis [110], and blood-based DNA methylation tests are thus now being explored to develop tests for early cancer diagnosis [111]. In lung cancer, methylation of ctDNA levels has emerged as a powerful biomarker able to plays a critical role in the detection and management of lung cancer across both late and early stages of the disease, in some case also before detectable radiological abnormalities, making them valuable for identifying cancer at its onset [112]. Recently, KMT2C (MLL3) aberrant promoter methylation was detected in the plasma cfDNA of NSCLC patients at both early and advanced stages, but not in the plasma of healthy individuals [113]. In advanced stage lung cancer, ctDNA methylation analysis continues to offer important diagnostic value but also serves in disease stratification, prognosis, and therapeutic decision-making. It can be used to monitor disease progression, assess treatment response, and detect MRD or relapse. Studies have shown that methylation markers in ctDNA correlate with tumour burden and can provide real-time insights into tumour dynamics [114]. The stability of methylated ctDNA fragments in circulation, even when ctDNA concentrations are low, makes methylation analysis particularly advantageous over mutation-based approaches, which often require a higher tumour DNA fraction for accurate detection.

## 5. Aberrant DNA Methylation in NSCLC Resistance

Aberrant DNA Methylation has been shown to play an important role in drug resistance in NSCLC, influencing responses to chemotherapy, target therapy, and immunotherapy.

Hypermethylation of the phosphodiesterase 3A (PDE3A) gene correlates with cisplatin resistance, and the epigenetic activation of forkhead box protein F1 (FOXF1) confers cancer stem cell properties to cisplatin resistance [115,116]. Wang et al. showed that methylation of the cadherin 13 (CDH13) promoter regulates cisplatin resistance in non-small cell lung cancer cells [117], while Liu et al. defined methylation of the P16 gene as associated with paclitaxel resistance, with demethylation reducing resistance in vitro [118].

With regards to resistance to TKI therapy, there are several evidence that have shown a role for methylation. Li et al. demonstrated that blocking DNA methylation of the epidermal growth factor receptor (EGFR) gene promoter can increase the efficacy of gefitinib [119], while another study explored the correlation between hypermethylation of the phosphatase and tensin homologue (PTEN) gene promoter and resistance to gefitinib or erlotinib [120]. Another gene whose hypermethylation has been associated with intrinsic resistance to EGFR-TKI is the homeobox B9 (HOXB9) gene [121]. Other methylated genes with low evidence associated with EGFR-TKI resistance in NSCLC are programmed cell death ligand (PD-L1) [121], gamma-aminobutyric scid type B receptor subunit 2 (GABBR2) [122], secreted frizzled related protein 5 (SFRP5) [123], death-associated protein kinase (DAPK) [124,125], Klotho (KL) and S100 calcium binding protein P (S100P) [126], secreted phosphoprotein 1 (SPP1) and CD44 [127], RASSF1A and growth arrest and DNA damage-inducible protein GADD45 beta (GADD45β) [128], Bcl 2 like protein 11 (BIM) [129], and cyclin dependent kinase 14 (CDK14) [130].

Regarding response to immunotherapy, Kim et al., starting from the analysis of fifteen immune-related pathways, developed a prognostic model based on eight genes to predict the clinical benefit of immunotherapy based on methylation patterns [131]. In a multicentre study, an epigenomic profile was established based on a microarray DNA methylation signature (EPIMMUNE) in a set of tumour samples from patients receiving nivolumab or pembrolizumab; the EPIMMUNE signature was associated with improved progression-free survival (PFS) (*p* = 0.0067) and OS (*p* = 0.0012) [132].

### 5.1. Aberrant Methylation and ctDNA Methylation Linked to KRAS G12Ci Resistance

DNA methylation has not yet been identified as a primary mechanism of resistance to Kirsten rat sarcoma (KRAS) inhibitors in NSCLC. Research in this field is ongoing and may provide new tools to understand treatment resistance. However, KRAS mutations were seen to be related to epigenetic changes and regulate many cellular processes. In some cellular model experiences, the KRAS G12V mutation is associated with significant changes in DNA methylation, affecting genes involved in cell development and differentiation, without any confirmation in tissues or liquid biopsy from lung cancer patients. The study was by Ben Yi Tew et al. and showed that KRAS G12V overexpression in isogenic lung cells (SAKRAS) led to over 50,600 differentially methylated CpG sites (DM CpGs) compared with non-transformed controls (SALEB). Further qRT-PCR analysis evaluating the mRNA expression of six genes with DM CpGs resulted in decrease levels of BRCA1 mRNA expression linked to promoter hypermethylation and increased NANOG and RELB expression in hypomethylation. The changes related to mutant KRAS expression include hypermethylation and hypomethylation of promoters of key genes, such as transcription factors, oncogenes, kinases, and differentiation regulators. Gene ontology evaluation obtained with the list of hypermethylated and hypomethylated gene promoters of SAKRAS cells revealed a transcriptional enhancement of genes related to development and differentiation. These epigenetic changes may contribute to tumorigenic potential and resistance to targeted KRAS treatments. The differential methylation induced by KRAS G12V appears to be stochastic and independent of canonical downstream effector signalling, such as the RAF-MEK-ERK cascade. ERK, indeed, represent the main effector pathway of KRAS and is involved in proliferation mechanism but seems to not be linked to methylation changes in KRAS signalling. Some evidence suggested the PI3K/AKT pathway as the effector of maintenance of epigenetic signature induced by KRAS mutation. These epigenetic changes may contribute to tumorigenic potential and resistance to targeted KRAS treatments [133].

CtDNA methylation was also suggested as an indirect biomarker for malignant KRAS mutated lung tumours and a good target for measuring ctDNA quantity. The referred study aimed to investigate the mutual agreement between methylation of homeobox A9 gene and KRAS mutation as an approach to detect ctDNA in advanced lung adenocarcinoma. The plasma sample from 48 lung tissue-matched non-malignant and malignant lung tissues from advanced disease stage patients and 100 plasma samples from healthy donors was analysed by bisulfite ddPCR for methylation and NGS for mutations. All tumours were positive for HOXA9 methylation, which was detected in matched 75% of plasma samples, with a good correlation with KRAS mutation in ctDNA (Spearman’s rho 0.83, *p* < 0.001), and both lesions were exclusive of the neoplastic condition [134].

### 5.2. ct(f)DNA Methylation Linked to TKI Resistance

To date, few studies have evaluated the potential of DNA methylation markers in ct(f)DNA in predicting resistance to TKIs (Table 2).

The first evidence derives from a monocentric study that evaluated the potential of parallel serial assessment of somatic mutation and methylation profile in monitoring the response to osimertinib in patients with stage IV lung adenocarcinoma and EGFR T790M mutation. A total of 85 longitudinal plasma samples were obtained from eight patients enrolled in the AURA17 clinical trial (NCT02442349). As a first result, a positive correlation was found between by-patient methylation level, calculated as methylation ratio (MR) and maximum allele fraction (maxAF) (*p* = 0.0002). In particular, methylation levels were higher in the plasma of patients with detectable somatic mutations than in patients without somatic mutations (*p* = 0.0003) and healthy controls (*p* = 0.0018). In addition, four trends in treatment response were detected; specifically, treatment efficacy was reflected by the significant reduction in methylation levels and maxAF, while a significant increase in these parameters correlated with impending morphological progression disease (PD). Finally, the results obtained from somatic mutation and methylation profiling in predicting early PD were compared; in particular, high levels of methylation and maxAF were observed in six and five patients in an average lead time of 3 and 1.9 months, respectively, before radiological PD [135].

Ntzifa et al. compared DNA methylation of nine genes in plasma cfDNA and paired circulating tumour cells (CTCs) from 42 NSCLC patients treated with osimertinib after progression to first- and second-generation TKIs. The investigated genes have been identified as epigenetically silenced in lung cancer based on previous evidence: RASSF1A, Ras-association domain family 10 (RASSF10), Wnt inhibitory factor-1 (WIF-1), adenomatous polyposis coli (APC), breast cancer metastasis suppressor 1 (BRMS1), DNA/RNA helicase Schlafen-11 (SLFN11), shisa family member 3 (SHISA3), retinoic acid receptor-beta (RARβ), and forkhead box protein A1 (FOXA1). Analyses were conducted at baseline and at the time of PD. A direct comparison of DNA methylation of these genes between plasma cfDNA and associated CTC samples (n = 70) revealed distinct methylation patterns, implying that CTCs and cfDNA provide complementary information. The authors discovered a substantial increase in methylation for at least one of these genes in PD when compared with baseline (*p* = 0.031). Furthermore, a trend was found for a statistically significant difference. Finally, a statistically significant difference in PFS was observed between patients who were positive for DNA methylation of at least one gene at PD and those who were negative (8.5 vs. 16.7 months; *p* = 0.066) [136]. Another study conducted by the same group of researchers found that in 27 patients with EGFR-mutated NSCLC receiving second-line osimertinib, there was an increase in methylation of at least one of these nine genes at PD compared with baseline in both plasma cfDNA and paired CTC analysis [137].

Shen et al. evaluated the correlation between WIF-1 methylation status and response to gefitinib in advanced stage EGFR-mutated NSCLC patients. The DNA methylation level of the WIF1 promoter was lower in the cfDNA of patients with partial or complete response to gefitinib, and patients with hypomethylated WIF1 had better PFS and OS [138].

A study conducted in Vietnam on 122 advanced EGFR-mutated patients who received EGFR-TKIs (gefitinib, erlotinib or afatinib) identified, by liquid biopsy (cfDNA), different patterns of DNA methylation and copy number alteration (CNA) changes among those who developed EGFR-dependent and -independent resistance mutations. Specifically, higher hypomethylation was found in cases with on-target resistances (T790M, EGFR amplification and co-occurrence of both), compared with those with off-target mutations (HER2 and MET amplification) (38.9% vs. 8.3%; *p* < 0.01). Genome-wide hipo-methylation and CNA correlated with the duration of drug response only in EGFR amplification cases. Furthermore, resistance mutations in EGFR were shown to accumulate abundant hypermethylation changes in the regulatory regions of suppressor genes, inducing their silencing and thus tumour progression [139].

A more recent study evaluated the role of DNA methylation in 103 patients with metastatic or locally advanced EGFR-mutated NSCLC, who had received afatinib. Assessment of the pre-afatinib cfDNA methylation profile showed that cases with a fatal outcome were accumulated in specific clusters. Genes for which cfDNA methylation levels were correlated with PFS were clustered in the cadherin, Wnt, and EGFR signalling pathways. Furthermore, pre-afatinib levels of centrosomal protein 170 (CEP170) and coiled-coil-helix-coiled-coil-helix domain containing 6 (CHCHD6) cfDNA methylation were associated with both PFS and OS. In addition, pre-afatinib and post-afatinib cfDNA methylation levels of solute carrier family 9 isoform A3 regulatory factor 2 (SLC9A3R2) and integrator complex subunit 1 (INTS1) correlated with bone metastasis. Finally, cfDNA methylation levels of two specific CpG sites (cg12721600 and cg05905155) prior to treatment has been shown to predict the response to afatinib, with a sensitivity of over 96% [140].

El Zarif et al. developed for the first time a non-invasive approach to detect small cell transformation (tSCLC) by epigenomic profiling of 1 mL of plasma in patients with EGFR-mutated lung adenocarcinoma progressing to EGFR-TKIs. Specifically, the researchers generated a multi-analyte SCLC risk score integrating three epigenomic features (histone modifications, DNA methylation, and chromatin accessibility) that demonstrated better accuracy than any single one of these analytes in discriminating between cfDNA samples from patients with tSCLC and EGFR-mutated adenocarcinomas (*p* = 0.0095; sensitivity and specificity of 89% and 91%, respectively). Furthermore, the authors observed in one patient that an increase in the cfDNA SCLC risk score preceded the clinical diagnosis of tSCLC by approximately three months [141].

The only study that evaluated cfDNA methylation profiling to monitor response in patients with anaplastic lymphoma kinase (ALK) rearrangements is that of Janke et al. The authors used a technique called cfMeDIP-seq (cell-free methylation DNA immunoprecipitation followed by high-throughput sequencing) to detect 5-mC signals, from blood plasma samples (n = 66) of 21 metastatic NSCLC lung adenocarcinoma patients treated with one or multiple lines of ALK-directed TKI therapy. The researchers generated a score (5-mC score) that showed high concordance with both chromosomal instability and EML4-ALK fusion abundances. In particular, higher 5-mC scores were associated with shorter OS (*p* = 0.025). Moreover, 5-mC scores have been shown to reflect therapy-associated tumour DNA dynamics in the plasma of these patients, playing a role in predicting response and eventual progression to target therapy. Longitudinal 5-mC scores were significantly elevated at PD compared with the therapy start (*p* = 0.0023). In addition, several cases of increased 5-mC scores before radiological progression were identified, with a median lead time of 89 days (0–345 days; *p* = 0.022) [142].

### 5.3. Aberrant Methylation and ctDNA Methylation Linked to ICI-Resistance

The clinical challenges in identifying NSCLC patients who will positively respond to immune checkpoint inhibitor (ICI) therapy and mechanisms of resistance still remain high unmet medical needs. The programmed death ligand 1 (PD-L1) expression in NSCLC drives the choice of ICIs with or without chemotherapy. However, PD-L1 expression does not be able to select all NSCLC patients who can benefit from ICI treatments. Thus, additional biomarkers that can improve the patient selection for ICIs are needed.

The correlation between epigenetic features and clinical benefit with PD-1 blockade has been retrospectively investigated from tumour tissue samples showing that the epigenetic setting of NSCLC tumours indicates which patients are most likely to benefit from ICI therapies [143]. A DNA methylation signature called the “EPIMMUNE” was found to be associated with clinical benefits in stage IV NSCLC patients who were treated with anti-PD-1 agents [132]. Moreover, pan-cancer analyses using TCGA (The Cancer Genome Atlas) data demonstrated that genomic global demethylation correlates with immune evasion signatures and affects the clinical benefits of immunotherapy with anti-PD-1/PD-L1 of lung cancer patients, despite the high mutation load [144].

Innovative ctDNA-based strategies from plasma to quantify the methylated loci, are being developed, to confirm the latest findings on tumour tissue biopsies.

5mC and 5hmC, epigenetic modifications in cfDNA, might influence response to therapy. 5hmC signatures in plasma-derived cfDNA, a stable epigenetic mark that originates from oxidation of 5mC, can be considered as a biomarker being positively correlated with early detection to treatment selection and response monitoring. A total of 151 blood samples were collected from 31 stage II-IV NSCLC patients at baseline and at multiple time points, with 4–6 weeks intervals, during anti-PD-1 therapy. In the 13 (42%) non-responder patients, the anti-PD-1 treatment induced changes in plasma cfDNA, 5hmC increased over epithelial to mesenchymal transition genes. In the 18 (58%) partial or complete responders to anti-PD-1 treatment, 5hmC accumulated over genes involved in immune activation such as interferon (IFN)-γ and IFN-α response, inflammatory response, and tumour necrosis factor (TNF)-α signalling, with the responses observed starting with the first cycle of treatment [145].

The 5hmC genome-wide profiling was performed on 85 plasma cfDNA samples from 83 NSCLC patients. A 5hmC predictive model was developed to quantify the 5hmC level and validate the model in both the validation and control sets. The low weighted predictive scores (wp-scores) had a median PFS of 7.6 months, which was considerably greater than 1.8 months for the high wp-scores in the validation (HR 0.12; *p* = 0.0012) and 14.9 against 3.3 months (HR 0.10; *p* = 0.00074) in the test sets. The objective response rates for low versus high wp-score were 75% versus 0.0% in the validation set (*p* = 0.019) and 80% versus 0.0% in the test set. Furthermore, wp-scores were substantially linked with PFS in individuals receiving single-agent ICI treatment (*p* < 0.05) [146].

In 20 advanced NSCLC patients, treated with anti-PD1-based immunotherapy, both baseline and follow-up blood draws, 4–10 weeks post-treatment initiation, were performed. Tumour methylation scores (TMSs), measured with an amplicon-based, multiplexed cfDNA assay, included the number of methylated molecules at more than 500 genomic locations that are hypermethylated in cancer tissue. The change in TMS, between the baseline and the 4–10 weeks post-treatment initiation samples, strongly correlated with immunotherapy response, as measured by real world PFS (*p* < 0.0001), and can be predictive of the response to ICI therapy [147].

The artificial intelligence (AI)-based methods have expanded the horizon for biomarker discovery, showing the power of integrating multimodal data from existing datasets to discover new meta-biomarkers for the prediction of benefit from immunotherapy [148]. The AI-based data with the above-mentioned studies provide proof-of-concept evidence that cfDNA signature is a strong biomarker for predicting ICI treatment response and resistance in NSCLC. This strategy needs validation in independent larger samples prospective studies.

## 6. Conclusions and Future Perspectives

The treatment of lung cancer patients has evolved mainly as a result of target treatments with TKIs and immunotherapy employing ICIs. Unfortunately, there are still significant problems with primary and secondary resistances to these therapies, and the molecular basis of these mechanisms is actually only partially clarified.

Up until now, the relevance of epigenetic changes in predicting the effectiveness of TKIs and ICIs has been underestimated, since they could represent key biomarkers to identify the best responders to TKI and immunotherapy, as well as targets to overcome the resistance to these treatments. Moreover, as the majority of epigenetic changes are reversible mediators of primary or secondary resistances, DNA methylation may pave the way for the utilization of combination therapies in patient subgroups with malignancies driven by epigenetics. DNA methylation analyses should also be incorporated into trials in conjunction with other established predictors to create a global predictive score in the context of personalized medicine and treatment optimization in order to advance and validate the usefulness of epigenetic markings in clinics. In the context of immunotherapy, it would likely be more effective to include epigenetic markers among a number of criteria in the immune-score computation. This would help identify patients who would require combination therapies as well as those who respond to ICIs. Furthermore, immunotherapy is not restricted to immune checkpoint inhibitors, and it is clear that epigenetic changes will affect responses to other promising therapeutic methods.

Anyhow, the routine feasibility and cost issues to expand the prognostic and predictive markers by including DNA methylation must also be considered. Currently, the investigation of epigenetic marks at a large scale is feasible for research purposes but could be difficult to validate in clinics due to the quantity and quality of tissue obtained in care settings. Monitoring ctDNA methylation has been shown to play an important role in overcoming this main issue, but it needs strong validation. Many technical and biological challenges remain at this moment, including technical variability in methylation detection platforms, potential confounding from non-tumour-derived methylation signals, and the need for standardization in clinical workflows.

Nothing is known about the best time window to perform cfDNA methylation analysis. In early-stage lung cancer, as well as during treatments, the amount of ctDNA in plasma may be extremely low. This can lead to false negatives or insufficient sensitivity for reliable detection. Moreover, the age-related and individual variability of DNA methylation profiles due to chronic inflammatory processes and other non-cancerous conditions could increase the risk of false positives. Moreover, DNA methylation patterns are often complex and dynamic, varying across tumour regions and between individuals, so advanced bioinformatics analysis is needed for the interpretations of results obtained from a plethora of not-jet-standardized or validated techniques currently used to analyse methylated DNA. As a consequence, results may lack reproducibility between different labs or clinical studies, which may not always be available in standard healthcare settings.

Despite substantial progress in cfDNA methylation profiling in lung cancer patients under targeted and immunotherapy observed in recent years, an extensive clinical validation on a larger scale is actually demanded before these methodologies can be reliably incorporated into routine diagnostic workflows.

Nevertheless, ctDNA methylation analysis represents a promising and increasingly validated tool for the early detection and clinical management of lung cancer across all disease stages.

## Figures and Tables

**Table 1:** Overview of cfDNA methylation profiling methods.

Method	Technology Category	Target	Advantages	Disadvantages	Input	Refs
WGBS	Bisulfite-based	Genome-wide	Base-resolution, unbiased coverage	DNA degradation, high cost	~125–250 pg	[39,40]
RRBS and cfRRBS	Bisulfite-based	CpG-rich regions	Cost-effective, single-base resolution	Requires high-quality DNA, limited coverage	≥10 ng	[43,44]
MCTA-Seq	Bisulfite-based	CGGCGG-rich CpGs	High sensitivity, very low input	Sequence bias, limited regions	~7.5 pg	[45,46]
Pyrosequencing	Bisulfite-based	Targeted CpGs	Quantitative, real-time analysis	Low sensitivity (<5%), not suitable for rare cfDNA	~10–50 ng	[48,77]
Targeted Bisulfite-Seq	Bisulfite-based	Selected regions	High resolution, scalable	Complex primer/probe design	50 ng	[51]
Methyl-Seq and cfMethyl-Seq	Bisulfite-based	CpG-rich cfDNA	Preserves cfDNA, UMIs for accuracy	Still requires bisulfite, potential loss	~5–10 ng	[52,53,54]
HM450K and HM850K arrays	Bisulfite-based	Predesigned CpG panel	Hotspot methylation with high accuracy	Low genome-wide coverage	~10 ng	[55,56]
qMSP	Bisulfite-based	Specific DMRs	Low input, high sensitivity	Locus-specific, limited multiplexing	~20–100 ng	[33,47]
ddMSP	Bisulfite-based	Specific DMRs	Ultra-sensitive, quantifies rare methylation events, suitable for cfDNA	Locus-specific, limited to known biomarkers, complex setup	~10–50 ng	[50]
Heatrich-BS	Bisulfite-based	CpG-dense cfDNA	Enrichment for CpG regions, efficient workflow	Not truly genome-wide	~5–10 ng	[57]
BSPP	Bisulfite-based	Specific loci (targeted CpGs)	High specificity via padlock probes, applicable	Complex design, requires optimization for each locus	~10–50 ng	[58,59]
MeDIP-Seq and cfMeDIP-Seq	Target enrichment-based	Methylated DNA	Preserves DNA, low input	Background noise, lower resolution	1–10 ng	[60,61,62]
5hmC-Seal	Target enrichment-based	5hmC	Detects epigenetic variants (5hmC), high sensitivity	Complex protocol, enrichment adds cost	~1–5 ng	[67,68,69]
MRE-Seq	Restriction enzyme-based	Unmethylated CpG sites	No bisulfite, methylation-sensitive digestion	Not ideal for cfDNA, low resolution	~10–50 ng	[70]
HELP	Restriction enzyme-based	Specific CpG sites	No bisulfite, relatively simple	Low genome coverage, not ideal for cfDNA	~10–50 ng	[71]
qPCR	Restriction enzyme-based	Probe-based PCR	Ultra-low input, fast turnaround	Very limited coverage, high false-positive/negative risk	~10–50 ng	[64,75,76]
ddPCR	Restriction enzyme-based	Probe-based PCR	High sensitivity and precision, ideal for rare allele detection in cfDNA	Limited to known loci, low multiplexing capacity	~10–50 ng	[72,73]

Abbreviations. WGBS, whole-genome bisulfite sequencing; RRBS, reduced representation bisulfite sequencing; cfRRBS, cell-free reduced representation bisulfite sequencing; CpG, cytosine–phosphate–guanine; MCTA-seq, methylated CpG tandem amplification and sequencing; cfDNA, cell-free DNA; Targeted Bisulfite-Seq, targeted bisulfite sequencing; Methyl-Seq, methylation sequencing; cfMethyl-seq, cell-free methylation sequencing; UMIs, unique molecular identifiers; HM450K, HumanMethylation450 BeadChip; HM850K, MethylationEPIC BeadChip; qMSP, quantitative methylation-specific PCR; ddMSP, digital droplet methylation-specific PCR; Heatrich-BS, Heatrich-bisulfite sequencing; BSPP, bisulfite padlock probe sequencing; MeDIP-seq, methylated DNA immunoprecipitation sequencing; cfMeDIP-seq, cell-free methylated DNA immunoprecipitation sequencing; 5hmC-Seal, 5-hydroxymethylcytosine Seal; MRE-Seq, methylation-sensitive restriction enzyme-sequencing; HELP, HpaII tiny fragment enrichment by ligation-mediated PCR; qPCR, quantitative polymerase chain reaction; ddPCR, digital droplet polymerase chain reaction. Ref, references.

**Table 2:** ct(f)DNA methylation and resistance in TKI-treated NSCLC.

References	Target	Population and Therapy	Biological Effects
[135]	*EGFR T790M* mutation	8 pts with IV stage LUAD osimertinib (post-1L)	Methylation levels are higher in ctDNA of pts with detectable somatic mutations than in pts without somatic mutations. The decrease in methylation levels and maxAF reflects treatment efficacy and the increase reflects PD.
[136]	*EGFR* mutation	42 pts with IV stage LUAD osimertinib (post-1L)	A significant increase in methylation is found for at least one of the 9 tested genes at PD compared to baseline. Difference trend in PFS is shown between pts who are positive for DNA methylation of at least one gene at PD and those who are negative.
[137,138]	*EGFR* mutation	27 pts with IV stage LUAD osimertinib (post-1L)	The increase in methylation is found for at least one of the nine tested genes at PD compared with baseline in both plasma cfDNA and paired CTC analysis.
[138]	*EGFR* mutation	Pts with IV stage LUAD gefitinib (1L)	Methylation level of *WIF1* promoter is lower in the cfDNA of pts with a complete or partial response to gefitinib. Pts with hypomethylated WIF1 have better PFS and OS.
[139]	*EGFR* mutation	122 pts with III-IV stage LUAD gefitinib, erlotinib, afatinib	Higher hypomethylation is found in cases with on-target resistances compared with those with off-target mutations. Hipo-methylation and CNA correlate with the duration of response only in *EGFR* amplification cases.
[140]	*EGFR* mutation	103 pts with III-IV stage LUAD afatinib (1L)	cfDNA methylation levels are correlated with PFS are clustered in the cadherin, Wnt and EGFR signalling pathways. Pre-afatinib levels of *CEP170* and *CHCHD6* cfDNA methylation are associated with both PFS and OS. Pre-afatinib and post-afatinib levels of *SLC9A3R2* and *INTS1* cfDNA methylation correlate with bone metastasis.
[141]	*EGFR* mutation	32 pts with IV stage LUAD EGFR-TKI	Histone modifications, DNA methylation, and chromatin accessibility allow discrimination between cfDNA samples from pts with tSCLC and EGFR-mutated LAUD.
[142]	*ALK*-rearranged	21 pts with IV stage LUAD crizotinib, ceritinib, alectinib, brigatinib, lorlatinib	Higher 5-mC scores is associated with shorter OS. 5-mC scores can predict treatment response and PD.

Abbreviations: LUAD: lung adenocarcinoma; ctDNA: circulating tumour DNA; cfDNA: circulating free DNA; CTC: circulating tumour cell; maxAF: maximum allele fraction; pts: patients; L: line; PD: progression disease; EGFR: epidermal growth factor receptor; *ALK*: anaplastic lymphoma kinase; TKI: tyrosine kinase inhibitor; *WIF1*: wnt inhibitory factor-1; PFS: progression-free survival; OS: overall survival; CNA: copy number aberrations; *CEP170:* centrosomal protein 170; *CHCHD6:* coiled-coil-helix-coiled-coil-helix domain containing 6; *SLC9A3R2:* solute carrier family 9 isoform A3 regulatory factor 2; *INTS1*: integrator complex subunit 1; tSCLC: small cell transformation; 5-mC: 5-methylcytosine.
